# Factors Associated with Attachment and Care Decisions

**DOI:** 10.1093/geroni/igab046.3600

**Published:** 2021-12-17

**Authors:** Kimberly Cassie

**Affiliations:** University of Oklahoma, Tulsa, Oklahoma, United States

## Abstract

It is widely accepted that remaining in the community for as long as possible is preferable to placement in a care facility. For many, this can only be realized with the support of a family caregiver. Previous research on the relationship between attachment and caregiving decisions is sparse, but tends to suggest there is a relationship between attachment and the decision to assume caregiving responsibilities, but more information is needed to better understand this unique relationship. This exploratory research seeks to address gaps in our understanding by asking is attachment related to the decision to care for a parent and what factors are associated with attachment. A convenience sample of 128 individuals caring for older parents was surveyed to answer these questions. Results indicate lower attachment related avoidance was associated with greater odds of caring for a recipient in the community rather than placing the recipient in a care facility. No relationship between attachment related anxiety and placement decisions was observed. Additionally, greater levels of attachment related avoidance were observed among caregivers reporting lower levels of filial responsibility, more adverse childhood experiences, less perceived support, and greater financial stability. Findings from this study can be used to support the development of interventions to strengthen attachment between adult children and their parents before care decisions are necessary.

